# Entropy Generation Minimization for Reverse Water Gas Shift (RWGS) Reactors

**DOI:** 10.3390/e20060415

**Published:** 2018-05-29

**Authors:** Lei Zhang, Lingen Chen, Shaojun Xia, Chao Wang, Fengrui Sun

**Affiliations:** 1 Institute of Thermal Science and Power Engineering, Naval University of Engineering, Wuhan 430033, China; 2 Military Key Laboratory for Naval Ship Power Engineering, Naval University of Engineering, Wuhan 430033, China; 3 College of Power Engineering, Naval University of Engineering, Wuhan 430033, China

**Keywords:** reverse water gas shift, tubular reactor, finite-time thermodynamics, entropy generation minimization, generalized thermodynamic optimization

## Abstract

Thermal design and optimization for reverse water gas shift (RWGS) reactors is particularly important to fuel synthesis in naval or commercial scenarios. The RWGS reactor with irreversibilities of heat transfer, chemical reaction and viscous flow is studied based on finite time thermodynamics or entropy generation minimization theory in this paper. The total entropy generation rate (EGR) in the RWGS reactor with different boundary conditions is minimized subject to specific feed compositions and chemical conversion using optimal control theory, and the optimal configurations obtained are compared with three reference reactors with linear, constant reservoir temperature and constant heat flux operations, which are commonly used in engineering. The results show that a drastic EGR reduction of up to 23% can be achieved by optimizing the reservoir temperature profile, the inlet temperature of feed gas and the reactor length simultaneously, compared to that of the reference reactor with the linear reservoir temperature. These optimization efforts are mainly achieved by reducing the irreversibility of heat transfer. Optimal paths have subsections of relatively constant thermal force, chemical force and local EGR. A conceptual optimal design of sandwich structure for the compact modular reactor is proposed, without elaborate control tools or excessive interstage equipment. The results can provide guidelines for designing industrial RWGS reactors in naval or commercial scenarios.

## 1. Introduction

The footsteps of human industrialization are always accompanied by massive levels of energy consumption. Excessive depletion of fossil fuels and the consequent CO_2_ emissions initiate a series of resource and environment crises, such as energy security, climate change and ocean acidification. Therefore, technologies related to CO_2_ emission reduction are flourishing in numerous scientific fields [1,2,3]. CO_2_ utilization by catalytic conversion with H_2_ from renewable energy is attractive, because this route can produce value-added chemical products with the detrimental greenhouse gas CO_2_ that are as cheap and abundant building blocks. This is the key to realizing a sustainable carbon cycle when the hydrogen is generated mainly from non-fossil energy [4,5,6]. Furthermore, the chemical conversion process is indeed a significant energy storage route which can convert solar, wind and floating nuclear into transportable, versatile and high energy-density hydrocarbon fuels despite the unfavorable energy balance [7]. The sea-based energy conversion process, a new method of fuel synthesis by CO_2_ and H_2_ extracted from seawater, has great significance for the security of offshore military energy and the development of alternative forms of energy [8]. The littoral fuel production in naval scenarios can significantly reduce the dependence on valuable fossil fuels, as well as the transportation costs and the resulting vulnerabilities from oil delivery. Research achievements related to sea-based fuel synthesis can also contribute to relieve extreme oil demand in remote fossil fuel poor locations [7,8,9]. 

Carbon dioxide can be hydrogenated into synthetic fuel by direct or indirect routes [10]. Because of the complex reaction mechanism and difficult activity and selectivity control, directly converting CO_2_ into a specific fuel is a challenging task [11], although some fuel type requirements can be achieved by designing bifunctional catalysts [12] and setting particular reaction conditions [13,14]. A typical indirect hydrogenation route, e.g., via producing syngas by RWGS (Reverse Water Gas Shift) to feed into a subsequent Fischer-Tropsch reactor has been considered as an alternative route for fuel synthesis both in naval scenarios and in commercial renewable fuel supply [10,11,15]. Endothermic RWGS thermodynamically favors a high reaction temperature, which can not only accelerate the chemical rate and improve equilibrium conversion, but also increase the energy consumption and costs of industrial applications [16,17]. RWGS has not been industrialized yet, the thermal design and optimization of the RWGS reactor using modern thermodynamic optimization theory is thus very important to technical applications on a large scale.

Finite-time thermodynamics (FTT) [18,19,20,21,22,23,24,25,26,27,28,29,30] is a new and developing multidisciplinary field in modern thermodynamics, where the time or rate factor neglected in classical thermodynamics is considered. FTT, also known as entropy generation minimization (EGM) [31,32,33,34,35] in engineering, can obtain the optimal performances and optimal configurations of various energy conversion devices and systems subjected to finite-time and/or finite-size, and the optimal results obtained are more powerful to guide thermal designs and optimizations of real-word devices. The wide applications of FTT in chemical reactions, heat and mass transfer processes have obtained many important theoretical achievements [36,37,38,39,40,41,42,43,44,45,46,47,48,49,50,51,52,53,54,55,56,57,58]. Månson and Andresen [36] firstly applied FTT to obtain the optimal paths of ammonia reactor with the maximum production rate as the objective function. Bak et al. [37], Chen et al. [38] and Wang et al. [39] investigated the optimal configurations of a generalized consecutive chemical reaction A⇌B⇌C and a more generalized one xA⇌yB⇌zC considering orders of the chemical reactions with the maximum yield of B [37,38] and the minimum entropy generation rate (EGR) [39] as objective functions, respectively. Chen et al. [40] obtained EGM analytical solutions of the combustion chemical reactions subjected to a given fuel conversion, which obey general rate equations. Wagner and Hoffmann [41,42] established endoreversible models of finite-rate chemical reactions [41] and used these extensions of endoreversible thermodynamics to investigate the maximum power output of a fuel cell [42]. Some scholars had studied the optimal configurations of the heat reservoir temperature profiles of the industrial reactors in depth by using the minimum EGR as an objective function, including the sulfur dioxide oxidation reactor [43], the tubular steam reformer [44,45] and the sulfuric acid decomposition reactor [46]. A set of guidelines for optimal reactors with the minimum EGR were also formulated based on the results obtained [45]. This equipartition of entropy production (EoEP), but also the equipartition of force (EoF) are good approximations to the state of the minimum EGR in the parts of some plug flow reactors with sufficient freedom. Ao et al. [47] obtained optimal temperature configurations of the tubular steam reformer with linear phenomenological heat transfer law $\left[q\propto \Delta (T^{-1})\right]$ based on EGM, which is different from those with the Newtonian heat transfer law [q∝Δ(T)] [44]. The chemical engineering processes, including ideal reactors, and the units both upstream and downstream, are also optimized with the minimum EGR as the objective function. Kingston and Razzitte [48,49] investigated the EGM of chemical process systems consisting of units of chemical reactors and other devices such as compressors and heat exchangers and so on, a case of dimethyl ether synthesis reactor was studied [49], and the optimal operating conditions were found. Wang et al. [50] studied the optimal path of sulfuric acid decomposition process with the maximum production rate of sulfur dioxide as the objective function. Zhang et al. [51,52] studied the power optimization of chemically driven heat engine combined FTT with probability theory. In terms of energy-saving optimization techniques of sea-based fuel synthesis, Chen et al. [53] analyzed and optimized the EGR of the removal process of CO_2_ from seawater by using the hollow fiber membrane contactor, the optimal concentrations configuration of CO_2_ for the minimum EGR were also obtained. Chen et al. [54] studied the reaction process of CO_2_ hydrogenation to light olefins by applying FTT theory, and the optimal design parameters for the minimum specific EGR (EGR averaged by the production rate of the target product) were obtained. 

In traditional chemical engineering, the optimal design of the industrial process units is driven by some objective functions related to cost, products and energy [55,56]. In the present work, the total EGR is considered as the objective function to perform the optimization work with specific constraints. It is essential to fully investigate the thermodynamic part before trading off the economic aspects by introducing more objective functions related to operation costs. EGR is a pure thermodynamic optimization indicator, which is the direct measure of the irreversibility. Optimization results based on EGM are independent of the current socio-political environment and markets, and the improvements for the chemical process units such as chemical reactors with the minimum EGR as objective function are related to the reduction of energy quality loss. This paper is devoted to exploring the potential for the EGR reduction in the RWGS reactor. FTT theory will be applied to find the operation state of the minimum EGR based on the formulation of optimal control problem. Moreover, the engineering application of the optimal reactor design will be discussed based on the optimization results.

## 2. The RWGS Reactor System

Considering the RWGS process in fixed-bed reactor

(1) $${\mathrm{CO}}_{2}+H_{2}⇌{\mathrm{CO}+H}_{2}O\;\Delta_{r}H>0$$

Except for the main product of CO in Equation (1), side reaction of methanation with CO is thermodynamically favored

(2) $$\mathrm{CO}+{3H}_{2}⇌{\mathrm{CH}}_{4}{+H}_{2}O$$

**Figure 1:**
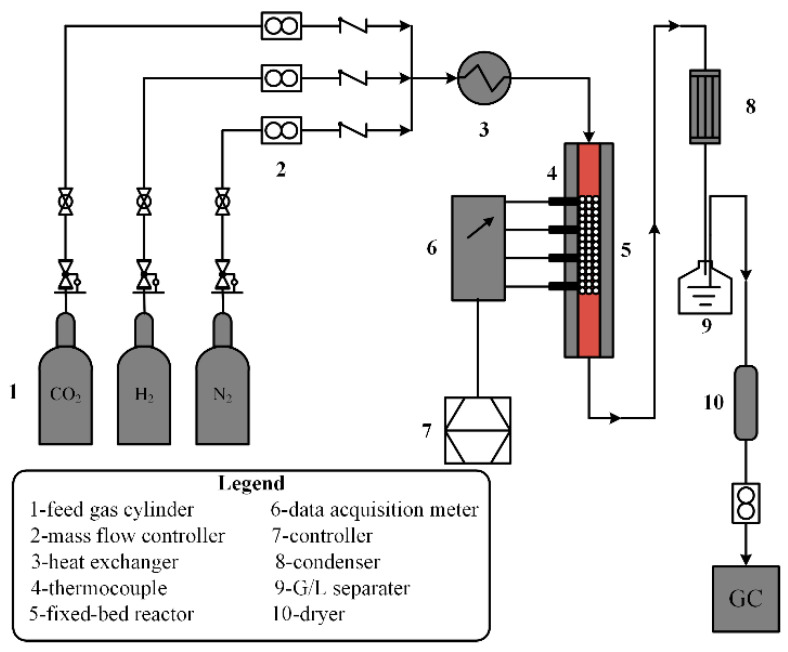
Schematic diagram of the lab-scale fixed-bed reactor.

A very high temperature and the resulting high energy losses and capital cost have to be maintained to suppress the formation of the by-product of CH_4,_ which is unfavorable for the downstream Fischer-Tropsch reactor [11,16]. Therefore, a high temperature-stable and high CO selective catalyst for RWGS reaction must be designed to achieve the technical and industrial requirements. A novel Pt-based catalyst designed by the research group of the author turned out to be a promising choice for RWGS reaction with almost no by-products CH_4_ in catalyst activity tests, a high CO yield and stable operation at high temperature. Selectivity and activity tests of this Pt-based catalyst were carried out in a lab-scale fixed-bed reactor as shown in Figure 1. The testing experiment was conducted in a stainless-steel reactor (790 mm length, 12 mm inner diameter) with an electrical heater. 1~3 mL of catalyst was packed in the middle isothermal section of the bed and the flow of the feed gases was controlled by the mass flow controllers. The experimental results showed that the CO selectivity reaches almost 100% in a typical reaction condition (pressure P = 1 atm, temperature T = 600 °C, gas hourly space velocity (GHSV) is 12,000 h^−1^, and feed gas molar ratio $H_{2}/{\mathrm{CO}}_{2}\;=\;2.5$). Therefore, only the central RWGS reaction (Equation (1)) is considered in the technical reactor modeling, while both the methanation side reaction (Equation (2)) and coking formation reaction are neglected. All compositions are in a gaseous state and are taken as ideal gases under the typical high-temperature operation condition.

### 2.1. The Technical Reactor Model 

**Figure 2:**
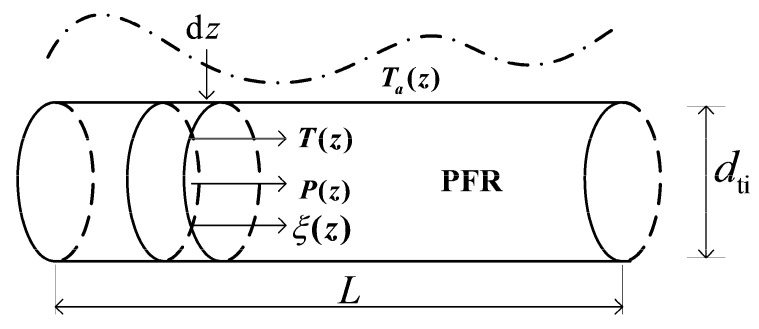
Schematic diagram of the one-dimensionless plug-flow technical reactor model.

The one-dimensional plug-flow assumptions are made to establish a simple technical reactor model as follows: (1) There are no gradients of state variables in the radial direction; (2) no dispersion or back-mixing occurs in the axial direction; (3) the chemical reactions are kinetically controlled, so all heterogeneous effects due to internal and external mass and heat transfer are disregarded. As shown in Figure 2, the reactor model is structured by the tube with inner diameter $d_{\mathrm{ti}}$ and length L. The spherical catalyst particles with diameter $d_{p}$ are distributed uniformly in the tube to establish a porous medium reaction channel, the void fraction of which is defined as ε. The heat is transferred in radial direction between the reaction mixtures and the heat reservoir outside the tube, and the temperature profile of the external heat reservoir is defined as $T_{a}(z)$. The operation state is characterized by the temperature of reaction mixtures T(z), pressure P(z) and chemical conversion ξ(z). The trajectories of state variables are changed along the axial coordinate z.

Given reaction (1), reactant CO_2_ is chosen as the reference component to express the reaction conversion which can keep track of all components along the reactor. The reaction conversion is expressed as

(3) $$\xi (z)=\left[F_{{\mathrm{CO}}_{2},0}-F_{{\mathrm{CO}}_{2}}(z)\right]/F_{{\mathrm{CO}}_{2},0}$$

where $F_{{\mathrm{CO}}_{2},0}$ and $F_{{\mathrm{CO}}_{2}}(z)$ are the molar flow rate in the inlet and the position z. Subscript “0” and “L” represent inlet and outlet states of the reactor.

The molar flow rates of all components $F_{k}$ can be expressed as functions of ξ

(4) $$F_{k}=F_{k,0}+F_{{\mathrm{CO}}_{2},0}\nu_{k}\xi$$

where $F_{k,0}$ and $\nu_{k}$ are the inlet molar flow rate and the stoichiometric coefficient of component k, respectively.

### 2.2. Conservation Equations

The conservation equations are used to govern the trajectories of the state variables. The energy balance in differential form is expressed as

(5) $$\frac{dT}{dz}=\frac{\pi d_{\mathrm{ti}}q-A_{c}\rho_{b}r\Delta_{r}H}{\sum_{k}F_{k}C_{p,k}}$$

where $A_{c}$ is the cross-sectional area of the reactor tube, r and $\Delta_{r}H$ are the reaction rate and enthalpy of reaction, respectively, $\rho_{b}$ is the catalyst bed density, and $C_{p,k}$ is the molar heat capacity of component k. $q\;=\;U(T_{a}-T)$ is the heat flux transferred perpendicular to the tube, which obeys Newtonian heat transfer law [q∝Δ(T)]. U is the overall heat transfer coefficient.

Ergun’s equation [57] is popular for modeling the pressure drop in the reactor, but it is only valid for the flow processes with relatively small Reynolds number, i.e., Re/(1−ε)<500. Re is the Reynolds number of packed bed, i.e., Re=vρdp/μ, where v is the superficial gas velocity, and ρ and μ are the density and viscosity of gas mixtures, respectively. The ideal gas law is used to calculate the gas velocity. The Reynolds number of the RWGS reactor is Re>3000 with the given working parameters in test calculation. Hick’s equation is thus used to model the momentum conservation [58].

(6) $$\frac{dP}{dz}=-6.8\frac{{\left(1-\varepsilon \right)}^{1.2}}{\varepsilon^{3}}{R_{e}}^{-0.2}\frac{\rho v^{2}}{d_{p}}$$

The mole balance based on the conversion ξ is given for the control volume as shown in Figure 2.

(7) $$d\xi /dz=A_{c}\rho_{b}r/F_{{\mathrm{CO}}_{2},0}$$

### 2.3. Chemical Reaction Rate

**Figure 3:**
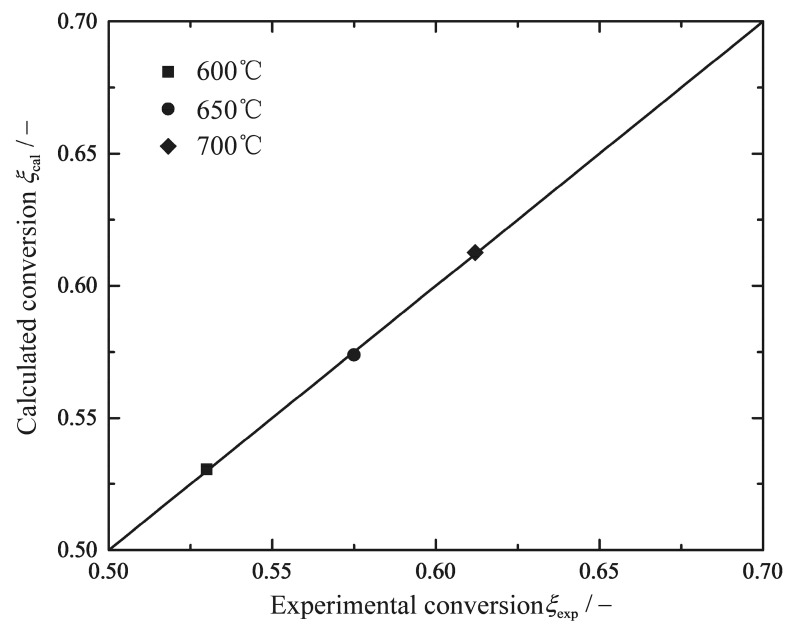
Comparison between experimental data and model predictions at 600 °C, 650 °C, and 700 °C (P = 1atm, GHSV = 12,000 h^−1^, $H_{2}/{\mathrm{CO}}_{2}=2.5$).

The chemical reaction rate is used to characterize the apparent performance of the catalyst and the rule how the contents of reactants and products change in the reactor. According to the irreversible thermodynamics, the fluxes in the transport phenomena should have a link with their corresponding driving forces. It is thus essential to use a reversible reaction rate expression to calculate the local EGR due to chemical reactions. A suitable reversible rate expression is used based on earlier work of Refs. [13,59,60]

(8) $$r=k\frac{P_{{\mathrm{CO}}_{2}}P_{H_{2}}-P_{\mathrm{CO}}P_{H_{2}O}/K}{P_{\mathrm{CO}}+a_{H_{2}O}P_{H_{2}O}+b_{{\mathrm{CO}}_{2}}P_{{\mathrm{CO}}_{2}}}$$

where $P_{k}$ is the partial pressure of component k, $a_{H_{2}O}$ and $b_{{\mathrm{CO}}_{2}}$ are adsorption coefficients representing inhibiting effects of H_2_O and CO_2_, respectively. K is the equilibrium constant which can be calculated by using standard Gibbs energy of reaction, $\Delta_{r}G_{T}^{0}$. k is the rate constant, a function of temperature T, can be expressed based on the Arrhenius equation

(9) $$k=Aexp[-E/\left(R_{g}T\right)]$$

where A and E are the pre-exponential factor and the activation energy, respectively. $R_{g}$ is the gas constant. The absorption coefficients for H_2_O and CO_2_ are kept constants and are taken from Riedel et al. [60] and Willauer et al. [59]. The missing kinetic parameters A and E need to be determined by fitting the experimental data from the catalyst performance tests in the present study. Table 1 lists the values of the kinetic parameters used herein, and Figure 3 shows the comparisons between experimental data and model predictions. It can be observed immediately that the simulation results with the calculated kinetic parameters are consistent with the experimental results.

### 2.4. Entropy Generation Rate

The local EGR of the one-dimensional plug-flow reactor is formulated according to the irreversible thermodynamics [46]

(10) $$\sigma =\pi d_{\mathrm{ti}}q(\frac{1}{T}-\frac{1}{T_{a}})+A_{c}v\left[-\frac{1}{T}\left(\frac{dP}{dz}\right)\right]+A_{c}\rho_{b}r\left(-\frac{\Delta_{r}G}{T}\right)$$

where σ contains contributions due to three transport phenomena: heat transfer, viscous flow and chemical reaction, respectively. Each term in the right side of Equation (10) is the couple of its flux and its corresponding driving force. The first term represents the pair due to heat transfer, where the flux is the heat flux q and the thermal driving force is $\Delta (1/T)=(1/T)-(1/T_{a})$. The second term is the couple for frictional flow; the flux is the gas velocity v and the viscous flow force is expressed as [−(1/T)(dP/dz)]. The last term is the sum of chemical reaction r and its conjugate chemical driving force $-\Delta_{r}G/T$, where $\Delta_{r}G$ is the Gibbs energy change of the reaction.

The total EGR $\Sigma_{\mathrm{tot}}$ is derived from the integral of σ over the axial coordinate z

(11) $$\Sigma_{\mathrm{tot}}=\int_{0}^{L}\sigma dz=\Sigma_{h}+\Sigma_{\mathrm{ff}}+\Sigma_{r}$$

where $\Sigma_{h}$, $\Sigma_{\mathrm{ff}}$ and $\Sigma_{r}$ are components due to heat transfer, viscous flow and the chemical reaction, respectively.

The total EGR can also be obtained from the entropy balance equation

(12) $$\Sigma_{\mathrm{tot}}=S_{\mathrm{out}}-S_{\mathrm{in}}-\pi d_{\mathrm{ti}}\int_{0}^{L}\frac{q(z)}{T_{a}(z)}dz$$

where $S_{\mathrm{in}}$ and $S_{\mathrm{out}}$ are the entropic contributions of the inlet and outlet gas flow, respectively. The last term in the right-hand side of Equation (12) is the entropy generation rate due to heat transfer.

## 3. Optimal Configurations of the RWGS Reactor

The optimization problem is to minimize the total EGR of the RWGS reactor with a given chemical conversion. The optimal temperature profiles or optimal configurations that result in the minimum total EGR are also obtained. Equation (11) is used as the objective function in the optimization, and Equation (12) is used to check the accuracy of the optimal solutions.

### 3.1. Parameter Settings of Reference Reactors

There are various heating or cooling designs for chemical reactors in engineering. The heat transfer strategies of linear, constant reservoir temperature and constant heat flux operations are commonly used in industry. For the reference reactor models, the three heat transfer strategies above are considered for the reactor cooling/heating system. The first reference design is the heat strategy in which reservoir temperature increases linearly along the axial coordinate, denoted as $T_{a}=\mathrm{linear}$; the reservoir temperature of the second case keeps constant, which can be achieved by using high-pressure boiling liquid and phase-change material as thermal fluid in the heat exchanges, and it is denoted as $T_{a}=\mathrm{const}$; the last case, denoted as q=const, is the operation under constant heat flux. The industrial and lab-scale tubular resistance furnaces with uniformly distributed heating wires are deemed to be operated under the constant heat flux mode. Reference parameters are set according to actual engineering standards, which are derived from Refs. [43,44,45,46,47,48,49,50,61]. The settings are listed in Table 2, the reservoir temperature profile is simulated as $T_{a}=[1073+100\times (z/L)]\;K$ for the $T_{a}=\mathrm{linear}$ reactor. For a fair comparison, the $T_{a}=\mathrm{const}$ reactor is modeled in such a way that its chemical conversion is equal to that of the $T_{a}=\mathrm{linear}$ reactor, in which reservoir temperature is thus set as $T_{a}=1136.6\;K$. Similarly, the heat flux for the q=const reactor is set as $q=17,936\;W\cdot m^{-2}$ to achieve the same conversion, in which temperature profiles can be obtained by solving conservation equations. The model has been solved as initial value problems by using the Matlab solver ***ode45***. All of the thermodynamic data are taken from Ref. [62].

### 3.2. Optimal Control Theory

The optimization problem is formulated based on optimal control theory. Firstly, the Hamiltonian H is given [43,45,46,63]

(13) $$H[x(z),\lambda (z),u(z)]=\sigma [x(z),u(z)]+\sum_{i=1}^{3}\lambda_{i}(z)f_{i}[x(z),u(z)]$$

where x=[T,P,ξ] are the state variable vector which are governed by conservation equations; $\lambda =[\lambda_{T},\lambda_{P},\lambda_{\xi }]$ represents three corresponding multiplier function vector for conservation equations $f_{i}$. The reservoir temperature $T_{a}(z)$ is considered as the control variable, i.e., $u(z)=T_{a}(z)$. And $T_{a}(z)$ is assumed to change continuously and freely over the entire reactor, their values keep finite and positive according to the engineering practice. The Hamiltonian herein is autonomous, which means the Hamiltonian of the system is independent of the evolution parameter, namely the axial coordinate z of the reactor. If the reactor length is specific, the Hamiltonian is constant over the entire reactor; and if the reactor length is free, the Hamiltonian must have a constant value of zero [43,64]. This property of the optimal solution can be used to check the validity of the results.

According to Pontryagin’s minimum principle [63], there are a set of necessary conditions, which are

(14) $$dT/dz=\partial H/\partial \lambda_{T}$$

(15) $$dP/dz=\partial H/\partial \lambda_{P}$$

(16) $$d\xi /dz=\partial H/\partial \lambda_{\xi }$$

(17) $$d\lambda_{T}/dz=-\partial H/\partial T$$

(18) $$d\lambda_{P}/dz=-\partial H/\partial P$$

(19) $$d\lambda_{\xi }/dz=-\partial H/\partial \xi$$

A control equation gives to formulate the link between state variables and multiplier functions

(20) $$T_{a}=\underset{T_{a}\in (0,+\infty )}{\arg\;\min}H\;z\in [0,L]$$

Equation (20) can be reduced to

(21) $$dH/dT_{a}=0$$

An algebraic restriction is introduced by solving Equation (21), as presented in Appendix A:

(22) $$T_{a}=T{\left(1+\frac{\lambda_{T}T}{\sum_{k}F_{k}C_{p,k}}\right)}^{-1/2}$$

Optimal control problem can be solved with certain boundary conditions. The production constraints in all optimizations are formulated as boundary conditions: the conversion is zero in the inlet of the reactor with fixed inlet compositions, and a specific value is given at the reactor outlet, i.e.,

(23) $$\xi_{0}=0\;\mathrm{and}\;\xi_{L}=\xi_{L}^{\mathrm{ref}}$$

where superscript “ref” represents the conditions in the reference reactor of $T_{a}=\mathrm{linear}$. The boundary conditions of the temperature and pressure can be adjusted according to the operation state of upstream and downstream equipment. The free boundary conditions, where the state variables change freely at the ends, are formulated in such a way that the corresponding multiple functions are zero at the ends. Therefore, other boundary conditions are expressed as

(24) $$\begin{array}{l} T_{0}=T_{0}^{\mathrm{ref}}\;\mathrm{or}\;\lambda_{T,0}=0\\ P_{0}=P_{0}^{\mathrm{ref}}\;\mathrm{or}\;\lambda_{P,0}=0\\ T_{L}=T_{L}^{\mathrm{ref}}\;\mathrm{or}\;\lambda_{T,L}=0\\ P_{L}=P_{L}^{\mathrm{ref}}\;\mathrm{or}\;\lambda_{P,L}=0 \end{array}$$

**Figure 4:**
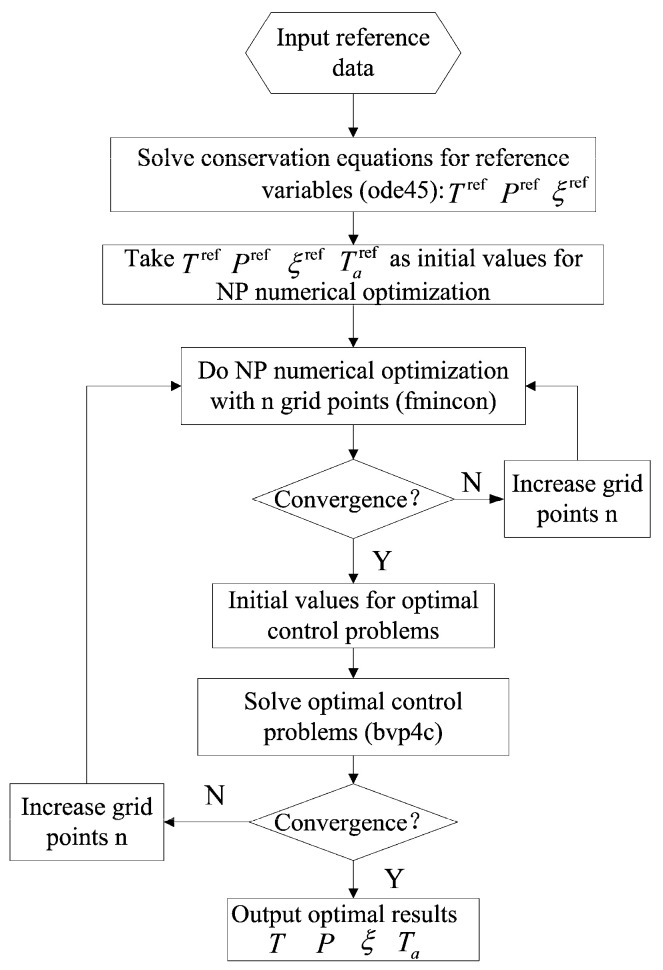
Program chart for the optimization algorithm.

In summary, the necessary conditions of the optimal control problem consist of six differential equations (Equations (14)–(19)), an algebraic equation (Equation (22)) and six boundary conditions given by Equations (23) and (24). The dynamic optimization problem is converted into a nonlinear two-point boundary value problem, which can be solved numerically by the Matlab solver ***bvp4c***. The ***bvp4c*** solver needs a good initial guess to converge by a collocation method. Ref. [47] found the optimal numerical solutions by the nonlinear programming (NP) method where a fine grid (300~400 points) must be used to find stable solutions. The algorithm introduced here uses the optimal solution by the NP method to create an initial guess for the ***bvp4c*** solver. The calculation results show that only a coarse grid (30~40 points) can possibly gain a quick convergence. An overview of the algorithm is shown in Figure 4. 

The calculations are arranged in the following five steps:

(1) Solve the reference reactor models to provide the boundary conditions for the following optimizations.
(2) The reactor model is optimized using $T_{a}(z)$ as the control variable with a fixed inlet temperature of the feed gas $T_{0}$, the optimal result is called “Case 1”.
(3) Take the reactor length L as an additional variable, the same optimization in step 2 is done between the range L∈[7 m, 8 m]. The optimal solution corresponding to the optimal reactor length $L^{\mathrm{opt}}$ is called “Case 2”.
(4) The following optimization is completed with $T_{a}(z)$ as the control variable, but with a free inlet temperature of the feed gas, the optimal result is called “Case 3”.
(5) Finally, the reactor length L is taken as an additional variable again with a free inlet temperature of the feed gas, the optimization work is performed repeatedly between the range L∈[0.1 m, 2 m]. The optimal solution corresponding to the optimal reactor length $L^{\mathrm{opt}}$ is called “Case 4”.

The boundary conditions used in all optimization cases are listed in Table 3, and the non-specific values are related to the free boundary conditions.

## 4. Numerical Examples and Discussions

As mentioned above, the conversions in all optimization cases are set as $\xi_{L}=0.48277$, which is derived from the solutions of the reference reactors.

### 4.1. Analyses of Numerical Results

Table 4 shows a detailed comparison of the three reference reactors and four optimal reactors. The reactor length, the total EGR and its contributions due to three transport phenomena, and the reduction in EGR compared with the reference reactor of $T_{a}=\mathrm{linear}$ are given. The EGR in q=const reactor is the minimum among the three reference reactors, oppositely, that in the $T_{a}=\mathrm{const}$ reactor is the maximum. The most contributions of irreversibilities for the reference reactors derive from heat transfer and chemical reactions. The reduction of EGR in all cases is a trade-off of the three transport phenomena of heat transfer, chemical reactions and viscous flow. The main reduction contribution in RWGS reactor is the heat component with the reservoir temperature profile as a control variable, which has a direct correlation to the heat transfer phenomenon. Only moderate EGR reductions up to 6.42% and 10.34% can be achieved with a fixed inlet temperature, by optimizing the reservoir temperature profiles and the reactor length. However, a further considerable reduction of up to 23.26% (14.05% for a specific reactor length) is achieved by setting the inlet temperature $T_{0}$ free. The reduction in Case 4 is due to a change in the components of the heat transfer and viscous flow. The contribution of the chemical reaction has increased by 86.09% because of a higher chemical reactivity caused by the increase of average temperature (see Figure 8). 

**Figure 5:**
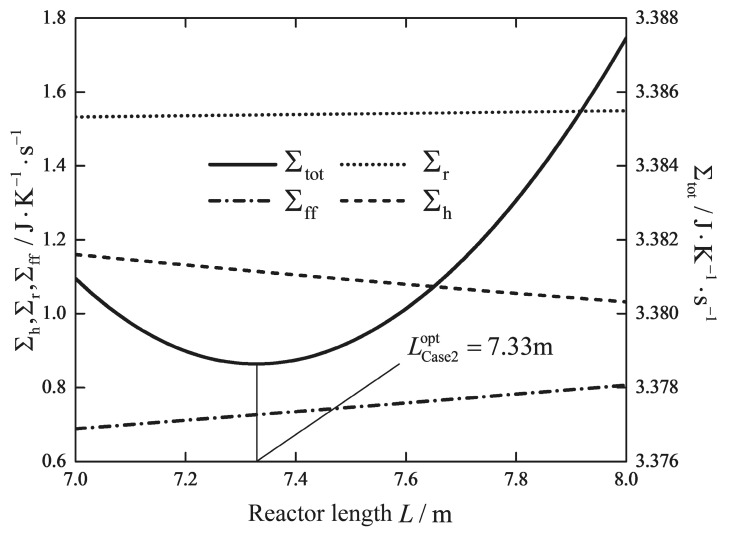
Variations of the EGR versus the reactor length L with a fixed inlet temperature $T_{0}$.

**Figure 6:**
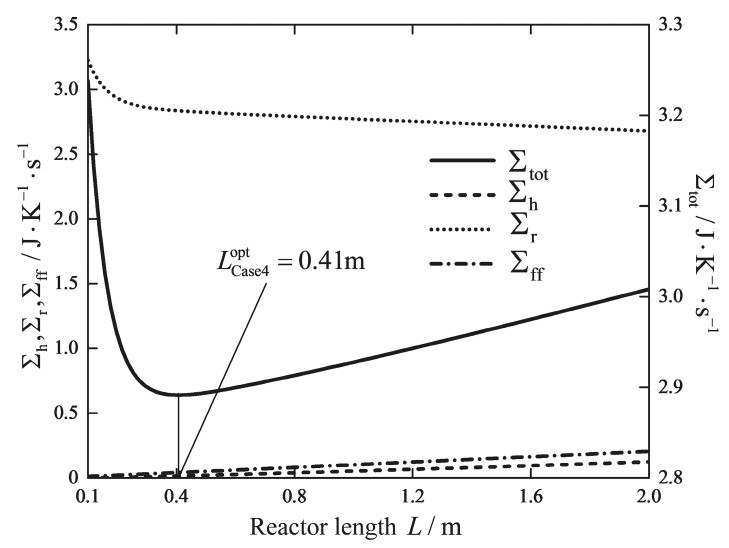
Variations of the EGR versus the reactor length L with a free inlet temperature $T_{0}$.

Figure 5 and Figure 6 show how the total EGR and its three contributions change with the reactor length for a fixed and free inlet temperature of the feed gas, respectively. It can be observed that an optimal reactor length exists for both cases. The range of the optimal reactor length for various boundary conditions is broad, and the optimal value depends very much on the boundary conditions. Therefore, the optimal reactor length of the RWGS reactor is closely dependent on the upstream and downstream settings. It is important to confirm the boundary conditions before performing the optimization. Increasing the reactor length beyond 5 m to the optimal value 7.33 m can further reduce the EGR due to heat transfer and chemical reaction, but a longer reactor leads to a trade-off in the entropic penalty resulted from higher pressure drop. It is interesting that the optimal reactor length is very much shorter than the reference length, changing from 5 m to 0.41 m. The compact modular chemical reactor design, which is built on a small scale, is ideal for sea-based fuel synthesis application with a low capital and operating costs [13,59,65]. The optimal solution of Case 4 implies that there is a significant potential to improve the exergy efficiency of the modular chemical reactor units, which have the equal production capacity as the industrial plant size design.

**Figure 7:**
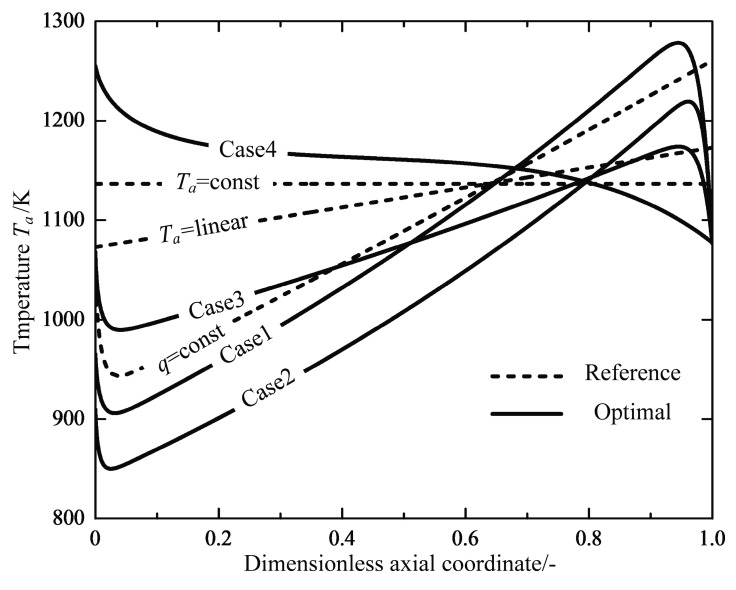
Variations of the heat reservoir temperature $T_{a}$ along the dimensionless axial coordinate.

**Figure 8:**
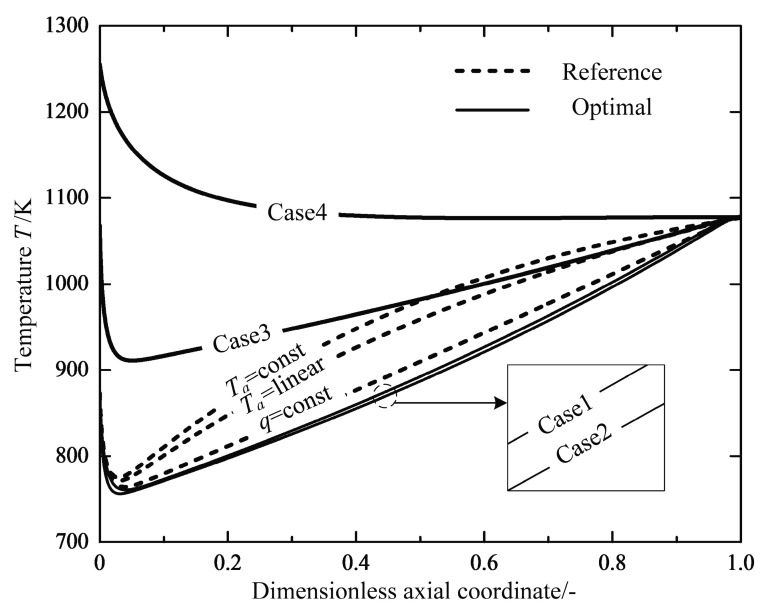
Variations of the temperature of reaction mixtures T along the dimensionless axial coordinate.

Figure 7 shows the profiles of the reservoir temperature for the reference reactors and optimal reactors, respectively. Through calculations, the averaged reservoir temperatures for three optimization cases are found to be lower than those of the reference reactors, except for Case 4. The reservoir temperature profiles follow the similar trend for the Cases 1, 2 and 3 reactors, they show rapid decreases near the inlet of the reactor, and are followed by nearly linear increases up to the maximum near the outlet, before they finally drop again to an approximately equal value. The profiles of the reaction mixture temperature are shown in Figure 8. It can clearly be seen that the starting temperatures in the Cases 3 and 4 reactors with a free inlet temperature are raised dramatically, which causes a larger reaction rate and more irreversibility losses due to the chemical reaction (see Table 4). It is interesting that the endothermic tubular steam reformer system under the operation state of the minimum EGR also has a higher inlet temperature than that of the reference reactor [44]. Therefore, it seems essential to preheat the feed gas in the endothermic reactors in order to reduce the total EGR. A heat exchanger should be included in the chemical system design to raise the reactant mixture temperature to the optimal initial inlet temperature obtained from optimal solutions, which was proposed by Kjelstrup et al. [64] and Wilhelmsen et al. [45]. 

**Figure 9:**
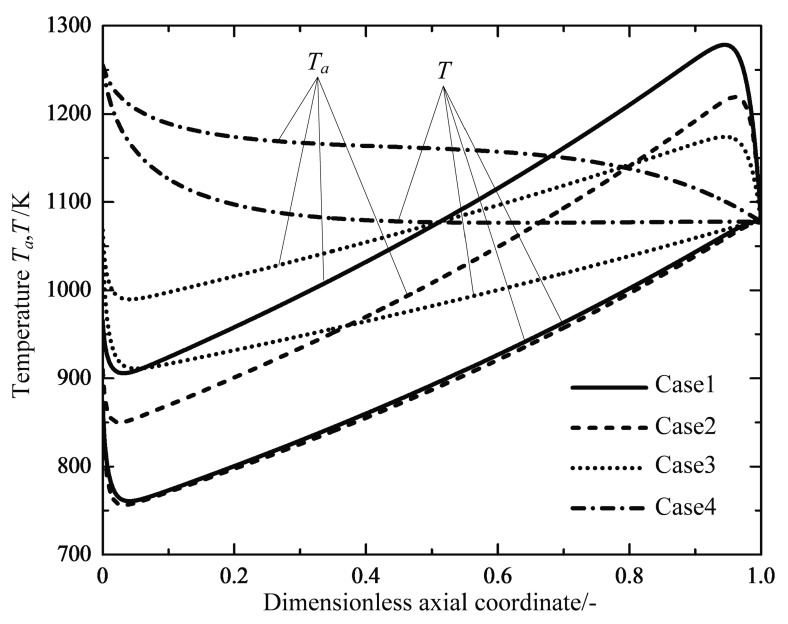
Optimal temperature configurations of the optimal reactors.

**Figure 10:**
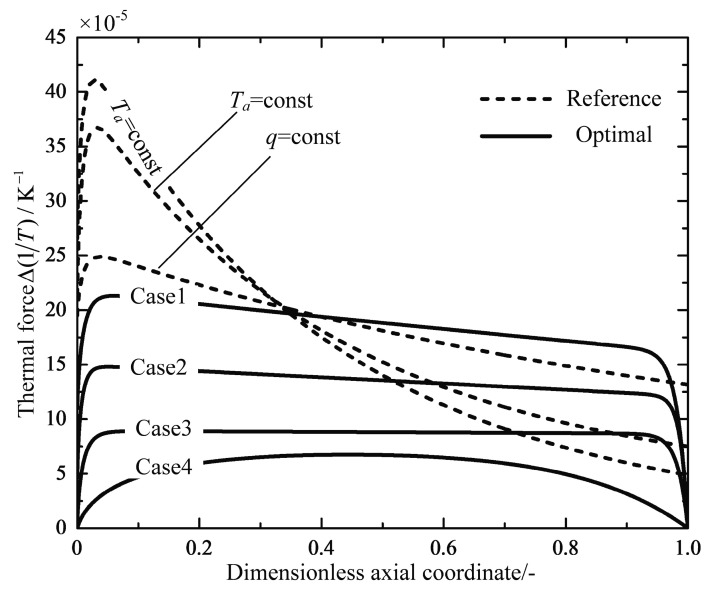
Variations of the thermal force Δ(1/T) along the dimensionless axial coordinate.

Figure 9 shows the optimal temperature profiles or configurations in the optimal reactors. There are subsections where the distance between the two temperature profiles are relatively constant for all optimal cases, which is referred to as a heat transfer mode [66]. The shape of the optimal temperature configuration with the free temperature boundary conditions at both ends is similar to the optimization results found by Nummedal et al. [44], which seem to be anti-symmetric closed profiles. The difference between the outer and inner temperatures are equal to zero at the ends of free boundary conditions. The free boundary conditions or natural boundary conditions in this optimal control problem introduce $\lambda_{T}=0$ to the optimal control Equation (22), which directly results in $T_{a}=T$ at the ends. It also implies that the optimal reactors have the potential of internal self-adjustment. In the early stage of the chemical reaction, an extremely high chemical force and the subsequent transformation between chemical energy and heat result in a rapid increase in thermal force (see Figure 10). Therefore, the optimal reactors spontaneously adjust to the zero-temperature difference in the inlet with the inlet free boundary condition. Similarly, another spontaneously adjustment appears in the last step of the reactions after the target of the production has been met.

**Figure 11:**
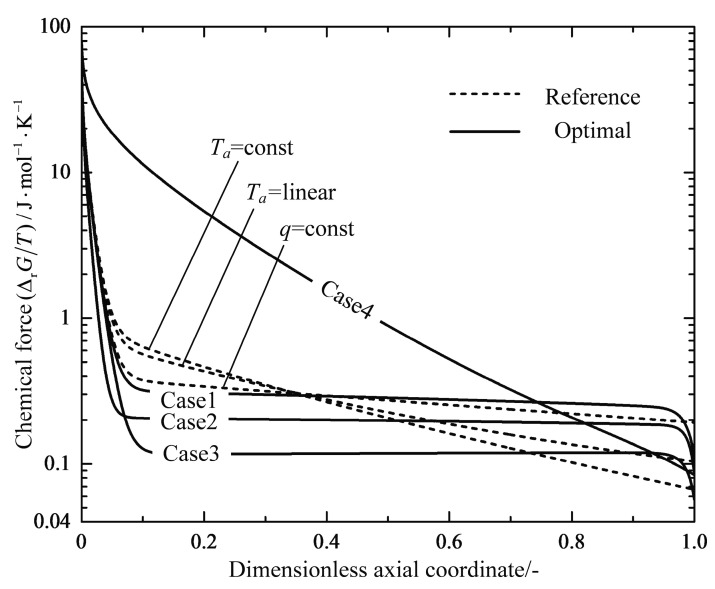
Variations of the chemical force $-\Delta_{r}G/T$ along the dimensionless axial coordinate.

Figure 10 and Figure 11 plot the profiles of the thermal forces and the chemical forces as functions of the dimensionless axial coordinate for the reference reactors and the optimal reactors, respectively. The thermal force profiles of the three reference reactors follow a similar trend, and decrease monotonously after rapid increases near the inlet of the reactor. The thermal force of the q=const reactor is the most evenly distributed compared with the other reference reactors. All of the four optimal reactors have major subsections of relatively constant thermal forces except for the inlet and outlet parts, and the Case 3 reactor indeed obtains the most evenly distributed thermal force profile. It can be seen in Figure 11 that the chemical forces in all cases decline dramatically from a high value at the reactor inlet to a rather constant value close to zero near the outlet of the reactor, which implies that the chemical reactions shift from the state of the large chemical driving force to the state close to the equilibrium, i.e., shift from a reaction mode to a heat transfer mode. The optimal reactors also have subsections of evenly distributed chemical force except for the Case 4 reactor.

**Figure 12:**
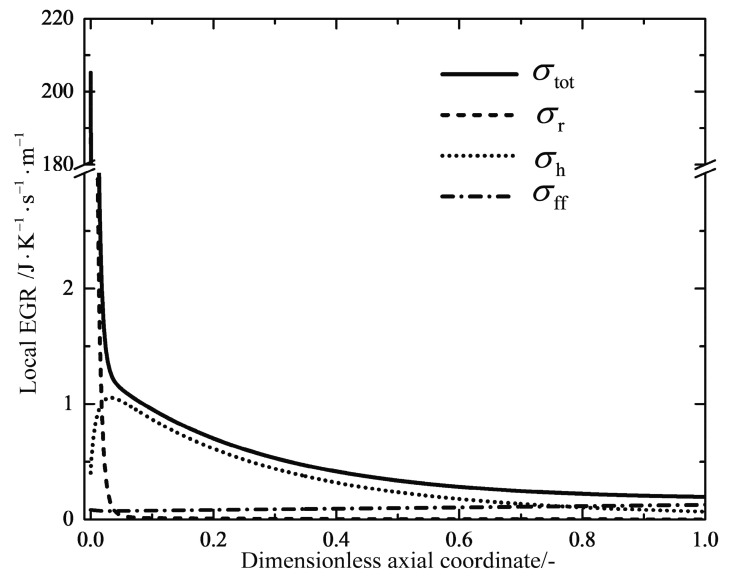
Variations of the total local EGR as well as the three components along the dimensionless axial coordinate for the reference $T_{a}=\mathrm{linear}$ reactor.

**Figure 13:**
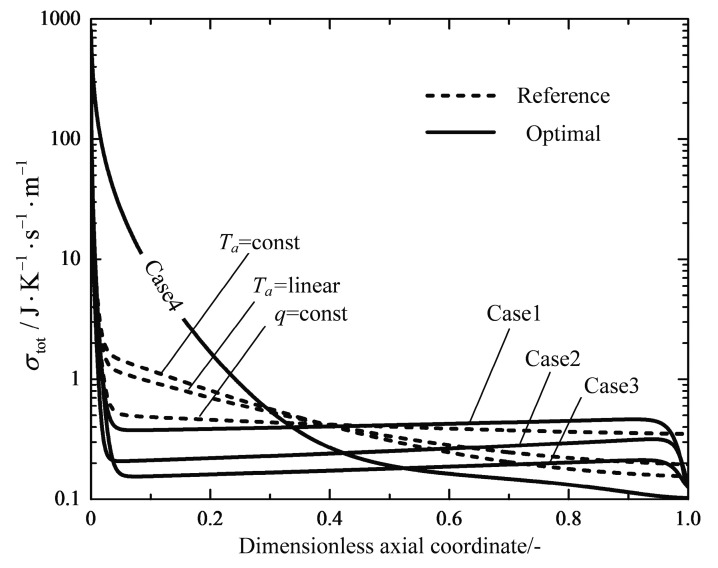
Variations of the total local EGR $\sigma_{\mathrm{tot}}$ along the dimensionless axial coordinate.

**Figure 14:**
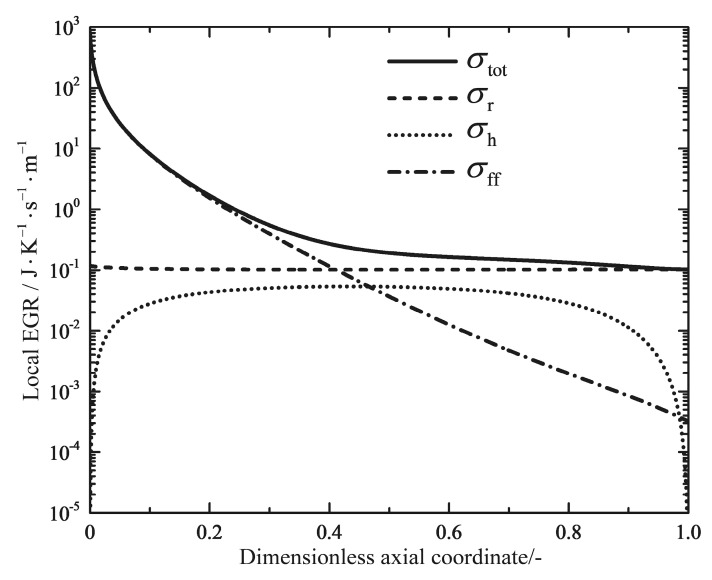
Variations of the total local EGR as well as the three components along the dimensionless axial coordinate for the Case 4 reactor.

Figure 12 shows the profiles of the total local EGR and its three components. $\sigma_{\mathrm{ff}}$, $\sigma_{h}$ and $\sigma_{r}$ are contributions due to the frictional flow, the heat transfer and the chemical reaction, respectively. It can immediately be seen that the component of the chemical reaction dominates only at the inlet of the reactor, which is caused by the high chemical driving force of the unreacted feed gas (see Figure 11). The heat transfer component shows a slight increase towards to a maximum near the inlet of the reactor; next it is followed by a gradual decrease to the outlet. Figure 13 shows profiles of the total local EGR for the reference reactors and the optimal reactors. The total local EGR in the reference reactors all have a very high initial value with steep drops and levels off to a more flat decrease right up to the end. It can obviously be observed that the EGM optimizations can obtain more evenly distributed total EGR profiles in most cases, except for Case 4. However, they still have very high initial values at the inlet, which are mainly caused by the chemical reactions and the high inlet temperature. A steep drop of the profile near the reactor outlet is related to the dramatic change in temperatures because of the boundary conditions of free outlet temperatures. It seems that EoEP is not appropriate for the Case 4 reactor according to Figure 13. Nevertheless, the local EGR due to heat transfer really has a relatively constant subsection for the Case 4 reactor as shown in Figure 14, which gives the profiles of the total local EGR and the three components for Case 4. EoEP is obeyed well in this respect.

**Figure 15:**
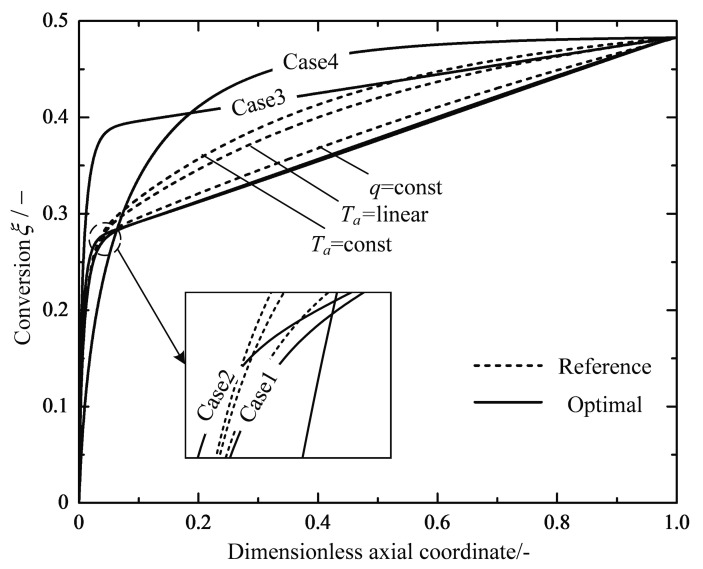
Variations of the chemical conversion ξ along the dimensionless axial coordinate.

Figure 15 shows how the chemical conversion changes with the dimensionless axial coordinate. All the profiles show different levels of steep increases close to the reactor inlet and level off to relatively flat increases. Almost 93.4% of the conversions can be achieved in the first third part of the Case 4 reactor. It can be inferred that the combination of a higher inlet temperature and a shorter reactor may achieve the production target with a considerable reduction in the total EGR. Nevertheless, the preferable design challenges the structural strength and the high-temperature performance of the catalyst. The other set parameters still need to be adjusted to conform with the design specification of compact modular reactors.

### 4.2. Engineering Applications

**Figure 16:**
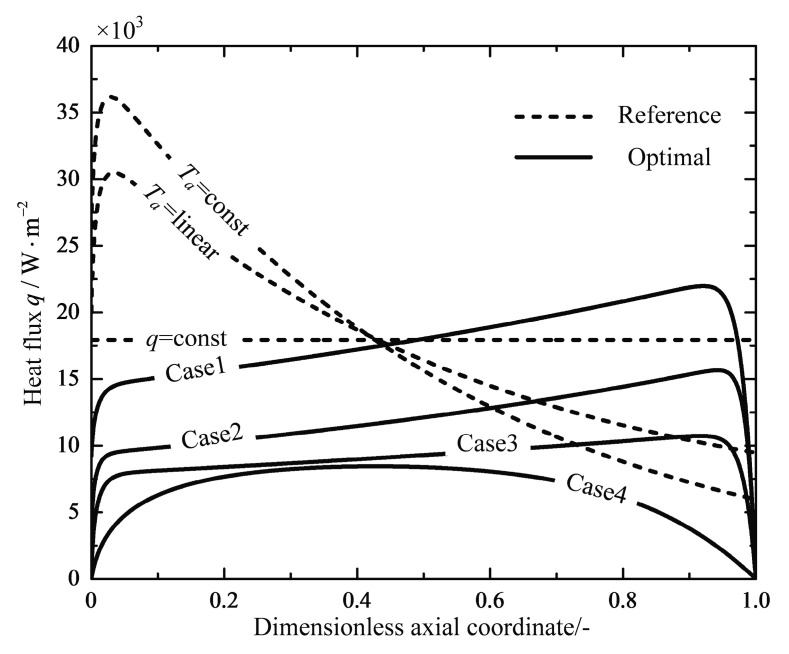
Variations of the heat flux q along the dimensionless axial coordinate.

**Figure 17:**
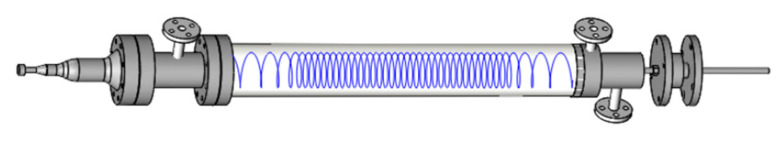
Conceptual design scheme of the optimal Case 4 reactor.

From the analyses above, it can be observed that the difference between the optimal path of the minimum EGR and the path of the reference reactor is significantly distinct. The optimal heat reservoir temperature profiles obtained with different boundary conditions can be realized by various engineering designs. Wilhelmsen et al. [45] formulated a set of guidelines for an exergy efficient reactor design. It indicated that an optimal design consists of an adiabatic pre-reactor followed by a reactor part operated in a heat transfer mode, where the distance between the reactant mixture temperature and the heat reservoir temperature keeps relatively constant. This part can be achieved by a counter-current heat exchanger. Another conceptual reactor design will be proposed according to the optimization results obtained in the present study. Figure 16 shows the profiles of the heat flux along the reactor length. It can be seen, as in Figure 16, that the optimal heat fluxes are relatively constant in the main part of the reactors except for the inlet and outlet of the reactors, and the optimal heat flux for the Case 4 reactor has the minimum average heat flux than the other optimal reactors, with a small-scale reactor length of 0.41 m and satisfactory 23.28% reduction in the total EGR. The heat flux is proportion to the amount of heating wires used in the design of the technical tubular resistance furnaces. Therefore, the optimal solution with a free inlet temperature favors a compact modular design of sandwich structure shown in Figure 17. The amount of heating wires is almost equal with relatively high values in the middle part after a gradual increase in the fifth of the reactor from zero, and finally drops gradually again to a final zero in the last fifth part. The considerable reductions in the total EGR and the amount of heating wires used can be achieved by using the optimal design instead of that of the reference q=const reactor. The proposed optimal reactor design with a compact and modular design concept, which provides flexible operations and competitive capital costs for littoral fuel synthesis process [13], gives a prototype to realize the optimal solutions that are solved based on an optimal control formulation, without elaborate control tools or excessive interstage equipment to model the optimal $T_{a}$-profile.

## 5. Conclusions

The technical RWGS reactor for the fuel synthesis process is studied, starting from establishing the reference reactors with the commonly used heat transfer strategies of the linear, constant reservoir temperature and constant heat flux. By solving the optimal control problem with different boundary conditions, the optimal configurations for the minimum total EGR involving heat transfer, chemical reaction and viscous flow are found by optimizing the heat reservoir temperature and the reactor length, subjected to given feed compositions and a fixed chemical conversion at the outlet of the reactor. The optimal numerical solutions are compared with those of the reference reactors. The results show that there is a great potential for the EGR reduction in the RWGS reactor. With respect to the typical reference reactor of $T_{a}=\mathrm{linear}$, up to 23.28% reduction in the total EGR can be achieved by the optimal design. Similar to the previous results of the minimum EGR in endothermic reactors, the irreversibility due to heat transfer can be reduced effectively by preheating the feed gas without considering the extra exergy costs spent upstream. A shorter reactor is suggested to do the same job with less EGR, which agrees with the development of compact modular reactor models. The optimal solutions related to small-scale reactor model is just an initial try to get a higher exergy efficiency, because more specification parameters should be adjusted according to engineering practice, and the influences of the design parameters on the EGR should be studied as well.

Two important theorems of EoEP (Equipartition of Entropy Production) [45,66,67] and EoF (Equipartition of Forces) [68] are possible to be used to judge the optimal operation of the minimum total EGR in the RWGS reactor. The optimal thermal force, chemical force and local total EGR profiles show large constant subsections except for the reactor inlet and outlet. But the theorems are only the post-optimization tools, not all the optimal cases obey the theorems exactly. The optimization work still needs to be done to find the optimal solutions for various situations [43]. 

Finally, an optimal design of sandwich structure for the technical compact modular reactor with electric heating wires has been presented based on the optimization results above. The heating wires should distribute loosely at the inlet and outlet of the reactor and the main subsection is distributed compactly. This set of the heating equipment provide a better choice in designing the RWGS reactor systems without further elaborate control tools or excessive equipment, compared with the general design of q=const. The results obtained herein can provide theoretical guidelines for real-word RWGS reactor design and optimization. 

## Figures and Tables

**Table 1:** Kinetic parameter values.

k (600 °C) mol/(s·g·MPa)	k (650 °C) mol/(s·g·MPa)	k (700 °C) mol/(s·g·MPa)	$a_{H_{2}O}$ -	$b_{{\mathrm{CO}}_{2}}$ -	A mol/(s·g·MPa)	E kJ/mol
0.0429	0.0547	0.0646	65	7.4	2.324	28.91

**Table 2:** Reference reactor parameters.

Parameter	Symbol	Value
Overall heat transfer coefficient	U	100 J∙m^−2^∙K^−1^∙s^−1^
Reaction mixture viscosity	μ	3.137 × 10^−5^ kg∙m^−1^∙s^−1^
Catalyst bed void fraction	ε	0.65
Catalyst pellet diameter	$d_{p}$	0.006 m
Inlet total molar flow	$F_{T,0}$	0.5 mol∙s^−1^
Catalyst bed density	$\rho_{b}$	1603 kg∙m^−3^
Reactor inner diameter	$d_{\mathrm{ti}}$	0.03 m
Reactor length	L	5 m
Inlet temperature of feed gas	$T_{0}$	873 K
Inlet total pressure	$P_{0}$	1 MPa
Inlet CO_2_ molar fraction	$y_{{\mathrm{CO}}_{2}}$	0.495
Inlet H_2_ molar fraction	$y_{H_{2}}$	0.495
Inlet CO molar fraction	$y_{\mathrm{CO}}$	0.005
Inlet H_2_O molar fraction	$y_{H_{2}O}$	0.005
Inlet reservoir temperature	$T_{a,0}$	1073 K
Outlet reservoir temperature	$T_{a,L}$	1173 K

**Table 3:** Boundary conditions for the optimal control problems.

Optimal Reactor Case	Case 1	Case 2	Case 3	Case 4
Inlet temperature	$T_{0}^{\mathrm{ref}}$	$T_{0}^{\mathrm{ref}}$	-	-
Inlet pressure	$P_{0}^{\mathrm{ref}}$	$P_{0}^{\mathrm{ref}}$	$P_{0}^{\mathrm{ref}}$	$P_{0}^{\mathrm{ref}}$
Inlet conversion	0	0	0	0
Outlet temperature	-	-	-	-
Outlet pressure	-	-	-	-
Outlet conversion	$\xi_{L}^{\mathrm{ref}}$	$\xi_{L}^{\mathrm{ref}}$	$\xi_{L}^{\mathrm{ref}}$	$\xi_{L}^{\mathrm{ref}}$
Reactor length	$L^{\mathrm{ref}}$	$L_{\mathrm{Case}\;2}^{\mathrm{opt}}$	$L^{\mathrm{ref}}$	$L_{\mathrm{Case}\;4}^{\mathrm{opt}}$

**Table 4:** Entropy generation rate (EGR) of the reference and optimal reactors.

Case	$T_{a}=\mathrm{linear}$	$T_{a}=\mathrm{const}$	q=const	Case 1	Case 2	Case 3	Case 4
Reactor length/m	5	5	5	5	7.33	5	0.41
$\Sigma_{r}$/(W/K)	1.5231	1.5308	1.5120	1.5068	1.5377	2.3475	2.8344
$\Sigma_{\mathrm{ff}}$/(W/K)	0.4952	0.5037	0.4762	0.4698	0.7267	0.5200	0.0422
$\Sigma_{h}$/(W/K)	1.7501	1.9029	1.5634	1.5497	1.1143	0.3716	0.0148
$\Sigma_{\mathrm{tot}}$/(W/K)	3.7684	3.9374	3.5516	3.5263	3.3787	3.2391	2.8913
Reduction/%	-	-	-	6.42	10.34	14.05	23.28

## Appendix A

The algebraic restriction based on the optimal control formulation derives from letting the derivation of the Hamiltonian with respect to the control variable $T_{a}$ is equal to zero

(A1) $$dH/dT_{a}=0$$

The variable of the reservoir temperature is only present in the heat transfer term of the local entropy generation rate and the right side of the energy conservation equation. Omitting the irrelevant terms, the reduced Hamiltonian gives as follows

(A2) $$H(T_{a})=\pi d_{\mathrm{ti}}q(\frac{1}{T}-\frac{1}{T_{a}})+\lambda_{T}\frac{\pi d_{\mathrm{ti}}q}{\sum_{k}F_{k}C_{p,k}}$$

where the heat flux is modelled as Newtonian heat transfer law

(A3) $$q=U(T_{a}-T)$$

Introducing Equation (A3) into Equation (A2), the reduced Hamiltonian is

(A4) $$H(T_{a})=\pi d_{\mathrm{ti}}U(\frac{T_{a}}{T}+\frac{T}{T_{a}}-2)+\lambda_{T}\frac{\pi d_{\mathrm{ti}}U(T_{a}-T)}{\sum_{k}F_{k}C_{p,k}}$$

By omitting the irrelevant terms again, Equation (A4) is reduced to

(A5) $$H(T_{a})=\frac{T_{a}}{T}+\frac{T}{T_{a}}+\frac{\lambda_{T}T_{a}}{\sum_{k}F_{k}C_{p,k}}$$

The derivation of Equation (A5) with respect to $T_{a}$ is

(A6) $$dH/dT_{a}=\frac{1}{T}-\frac{T}{T_{a}^{2}}+\frac{\lambda_{T}}{\sum_{k}F_{k}C_{p,k}}=0$$

The optimal control $T_{a}$ can be obtained by solving Equation (A6)

(A7) $$T_{a}=T{\left(1+\frac{\lambda_{T}T}{\sum_{k}F_{k}C_{p,k}}\right)}^{-1/2}$$
